# The Role of Cytokines and Chemokines in Severe Acute Respiratory Syndrome Coronavirus 2 Infections

**DOI:** 10.3389/fimmu.2022.832394

**Published:** 2022-04-07

**Authors:** Ren-Jun Hsu, Wei-Chieh Yu, Guan-Ru Peng, Chih-Hung Ye, SuiYun Hu, Patrick Chun Theng Chong, Kah Yi Yap, Jamie Yu Chieh Lee, Wei-Chen Lin, Shu-Han Yu

**Affiliations:** ^1^Cancer Center, Hualien Tzu Chi Hospital, Buddhist Tzuchi Medical Foundation, Hualien, Taiwan; ^2^School of Medicine, College of Medicine, Tzu Chi University, Hualien, Taiwan; ^3^Institute of Biotechnology, National Taiwan University, Taipei, Taiwan; ^4^University of California, Los Angeles, Los Angeles, CA, United States

**Keywords:** COVID-19, cytokines, chemokines, infection, diagnostic markers

## Abstract

Severe acute respiratory syndrome coronavirus 2 (SARS-CoV-2) has resulted in countless infections and caused millions of deaths since its emergence in 2019. Coronavirus disease 2019 (COVID-19)-associated mortality is caused by uncontrolled inflammation, aberrant immune response, cytokine storm, and an imbalanced hyperactive immune system. The cytokine storm further results in multiple organ failure and lung immunopathology. Therefore, any potential treatments should focus on the direct elimination of viral particles, prevention strategies, and mitigation of the imbalanced (hyperactive) immune system. This review focuses on cytokine secretions of innate and adaptive immune responses against COVID-19, including interleukins, interferons, tumor necrosis factor-alpha, and other chemokines. In addition to the review focus, we discuss potential immunotherapeutic approaches based on relevant pathophysiological features, the systemic immune response against SARS-CoV-2, and data from recent clinical trials and experiments on the COVID-19-associated cytokine storm. Prompt use of these cytokines as diagnostic markers and aggressive prevention and management of the cytokine storm can help determine COVID-19-associated morbidity and mortality. The prophylaxis and rapid management of the cytokine storm appear to significantly improve disease outcomes. For these reasons, this study aims to provide advanced information to facilitate innovative strategies to survive in the COVID-19 pandemic.

## Introduction

Severe acute respiratory syndrome coronavirus 2 (SARS-CoV-2) and its associated pathology, coronavirus disease 2019 (COVID-19) are of particular concern because of the global pandemic they have unleashed. COVID-19 continues to challenge medical health systems globally, and as the scenario is inevitably worsening (1, 2), developing prophylactic and therapeutic approaches is urgent. The number of cases requiring intensive care has emerged as a critical point in the epidemic. Therefore, it is important to understand in more detail the pathophysiology underlying severe disease, particularly the factors that drive severe lung pathology after infection with the highly pathogenic human coronaviruses (3).

The hyperactive host immune response to SARS-CoV-2 infection leads to an exaggerated inflammatory reaction. Elevated circulating levels of interleukin (IL)-1β, IL-2, IL-6, IL-7, IL-8 (CXCL8), IL-9, IL-10, IL-17, IL-18, IL-22, IL-33, granulocyte-colony-stimulating factor (G-CSF), granulocyte-macrophage colony-stimulating factor (GM-CSF), interferon (IFN)-γ, tumor necrosis factor (TNF)-α, chemokine (C-X-C motif) ligand (CXCL)10, monocyte chemoattractant protein 1 (CCL2 or MCP-1), macrophage inflammatory protein 1 (MIP-1)A (CCL3), CX3CL1 and MIP-1B (CCL4) (4, 5) have been reported in patients with COVID-19, particularly in those admitted to the intensive care unit (ICU). The overexpression of cytokines and chemokines triggers the severe cytokine release syndrome (CRS), which increases the severity of the disease even more (6, 7). Moreover, samples of the bronchoalveolar lavage fluid (BALF) of patients with COVID-19 show the accumulation of various immune attractant chemokines, including C-C motif chemokine ligand (CCL)2, CCL3, CCL4, CCL7, CCL8, CCL20, CXCL6, and CXCL11 (4, 8, 9). Many of these chemokines are secreted by monocytes or macrophages (4, 5, 10). Researchers that have analyzed cytokine profiles in patients with COVID-19 suggest that cytokine storms correlate directly with lung injury, multiorgan failure, and unfavorable prognosis in severe COVID-19 (11).

Using available data from experimental and clinical studies, this review aims to provide up-to-date knowledge on cytokine and chemokine secretions induced by host immune responses in COVID-19 as well as on cytokine storm and related pathophysiological features. Identifying the distinct cytokine/chemokine profile(s) and pathophysiological features of COVID-19-induced cytokine storm has practical implications as it can predict clinical deterioration, e.g., the requirement of intubation or mortality. Understanding the nature of the immune response that can lead to recovery from severe COVID-19 remains key to developing effective treatments (12).

## Cytokine Secretion Induced by SARS-CoV-2 Infection

SARS-CoV-2 infects cells by attaching itself to the angiotensin-converting enzyme 2 (ACE2) (13) and/or type II transmembrane serine protease (TMPRSS2) receptors (14). Subsequent viral replication and release cause the host cell to undergo pyroptosis and unleash pathogen-associated molecular patterns (PAMPs). In the first few days following infection, innate immune cells, including macrophages, dendritic cells (DCs), neutrophils, and natural killer (NK) cells, are activated when they recognize viral PAMPs (15). Next, PAMPs trigger the production of several proinflammatory cytokines and chemokines, including TNF, IL-6, type-1 IFN, CCL2, CCL3, CCL4, and CXCL10 (IFN-γ-induced protein 10 kDa [IP-10]) (16, 17), which lead to the recruitment of monocytes, macrophages, and T cells to the infection sites. These processes promote further inflammation and recruit NK cells (3) and T cells, which produce more IFN-γ (18). Innate antigen-presenting cells, such as DCs and macrophages, at the infection site also present viral antigens to virus-specific T cells. This leads to the activation of the body’s adaptive immunity, which is mediated by virus-specific B (humoral immunity) and T cells (cellular immunity) (10, 19).

When SARS-CoV-2 binds to the ACE2 receptor to enter the target cell, the renin-angiotensin system (RAS) is activated, and angiotensin II (Ang II) levels increase in circulation (20, 21). Studies have shown that the ACE2-Ang II axis can induce the infiltration of macrophages and secretion of certain cytokines, including IL-6, CCL2, vascular cell adhesion molecule 1 (VCAM-1), and E-selectin, to induce endothelial dysfunction, thrombin formation, and impaired fibrinolysis (20, 22–24). In addition to the ACE2-Ang II axis, viral protein Nsp5 and spike (S) in SARS-CoV-2 can potently induce inflammatory cytokines and chemokines IL-1β, IL-6, TNF-α, IL-2, CXCL1, CXCL2, and CCL2 expressions in monocytes/macrophage through NF-κB signaling pathways (25, 26).

Both aspects of adaptive immunity, i.e., cell-mediated immunity and humoral immunity, have critical roles in COVID-19 (27). Lower absolute numbers of T lymphocytes (CD4^+^ and CD8^+^ T cells) occur in both mild and severe infection, with a more notable decrease in severe cases. The decrease in IFN-γ expression by CD4^+^ T cells is also greater in severe cases than in moderate ones (28). Moreover, pyroptotic cell death can lead to higher serum IL-1β levels and trigger neutrophil migration and T cell activation (29, 30). Indeed, neutrophil cytotoxicity releases leukotrienes and reactive oxygen species, thereby triggering acute lung injury (ALI) and a cytokine storm. Neutrophils may also lead to endothelial injury, which can further promote systemic virus dissemination (17, 30–32). Furthermore, increased serum levels of IL-1β, IFN-γ, CXCL10, and CCL2 strongly point toward the activation of T helper 1 (Th1) cell function (33, 34). Thus, inadequate innate and adaptive antiviral defenses, in addition to high proinflammatory cues, lead to multiorgan damage (10, 35).

To further illustrate the effect of SARS-CoV-2 on cytokine and chemokine production, 27-plex cytokine assay panels and the Bio-Plex 200 system were used to analyze plasma cytokine and chemokine levels during the acute phase of the illness, and available results indicate that the plasma concentrations of specific inflammatory cytokines and chemokines highly correlate with the severity of COVID-19 disease course. In particular, the expression levels of IL-2, IL-7, IL-10, GCS-F, CXCL10, CCL2, CCL3, and TNF-α were significantly higher in ICU patients than in non-ICU patients (33). By contrast, other cytokines, including IL-1β, IL-1RA, IL-7, IL-8, IL-9, IL-10, basic fibroblast growth factors (FGF), GCS-F, GM-CSF, IFN-γ, CXCL10, CCL2, CCL3, CCL4, platelet-derived growth factor subunit B (PDGFB), TNF-α, and vascular endothelial growth factor-A (VEGF-A), showed higher expression in both ICU and non-ICU patients with COVID-19 (33, 36, 37). Of note, the expression of some cytokines, such as IL-5, IL-12, p70, IL-15, CCL5, and eotaxin, were similar in healthy adults and SARS-CoV-2-infected patients (5, 6, 28, 33, 38–40).

Due to the importance of cytokines in COVID-19 disease development, several clinical trials have been focusing on cytokine modulation. According to the ongoing clinical trials, many potential COVID-19 therapeutic drugs, such as anakinra (IL-1 receptor antagonist) (13), siltuximab (IL-6 neutralization) (41), sarilumab (IL-6 receptor antagonist) (41), tocilizumab (IL-6 receptor antagonist) (42), secukinumab (IL-17 neutralization) (43), baricitinib (Jak inhibitor) (44), IFN-β-1α (inducing type I IFN-stimulated genes) (45), recombinant human IFN drugs (46), eculizumab (inhibition of complement activation) (47), G/10 (innate response regulation) (48), CD24F (innate response regulation) (48), anti-GM-CSF monoclonal antibody (49) and NKG2D-ACE2 CAR-NK cells (50) have been applied to COVID-19 patients to boost their immune system, inhibiting viral replication or cytokine storms (42, 44, 48, 51–56). Accordingly, these trials indicated that some cytokines, such as IL-1 (57), IL-6 (58), TNF (57), IFN (59), and GM-CSF (57), could be potential targets to develop as diagnostic markers and personalized drug targets (58). In addition to the potential targets, there are a number of small-molecule drugs under clinical trials to benefit COVID-19 patients. Paxlovid, a ritonavir-boosted protease inhibitor, is the first oral antiviral drug to receive FDA granted EUA in December of 2021 (41). Both remdesivir (60) and molnupiravir (61, 62) can inhibit viral replication by inhibiting RNA-dependent RNA polymerase (RdRp) (60–64).

**Figure 1:**
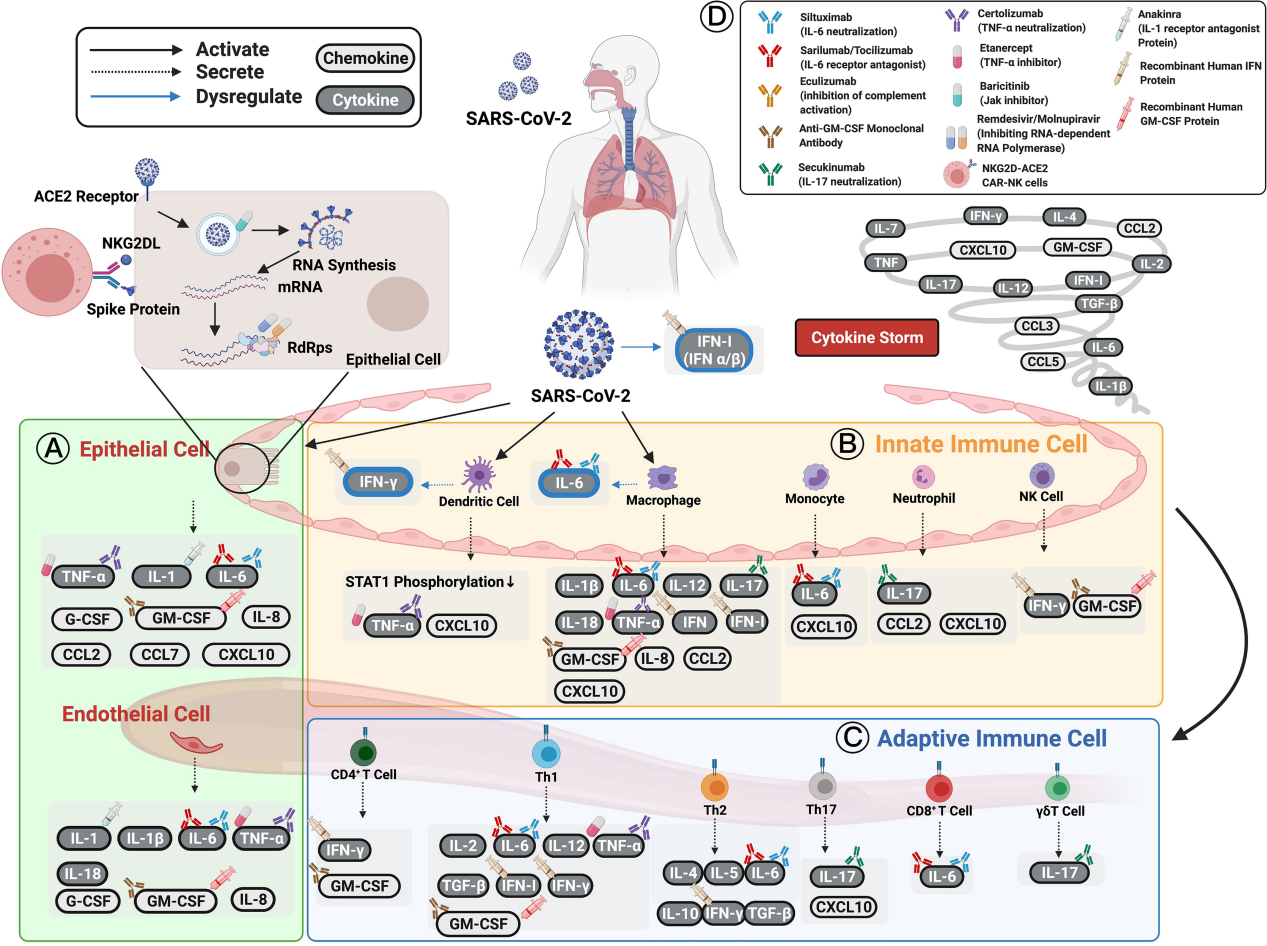
Cytokines and chemokines expression induced by SARS-CoV-2 in patients with COVID-19. **(A)** Epithelial cells have been shown to express TNF-α, IL-1, IL-6, and other chemokines (e.g., IL-8, CCL2, CCL7, CXCL10, G-CSF, and GM-CSF) as well as initiate immune responses following infection. **(B)** Innate immune cells, including dendritic cells, macrophages, circulating monocytes, neutrophils, and NK cells, are activated by SARS-CoV-2 infection to secrete various cytokines and chemokines to enhance both innate and adaptive immune cells. **(C)** Adaptive immune cells consisting of functional CD4^+^ T cells, Th1 cells, Th2 cells, Th17 cells, CD8^+^ T cells, and γδT cells defend against SARS-CoV-2 infection. The cytokines and chemokines expressed by adaptive immune cells interact in a positive feedback loop to strengthen innate immune responses. The cellular origin of each cytokine and its regulatory roles are shown in figure and **Table 1**. The persistent escalation of these responses leads to uncontrolled cytokine and chemokine expression, resulting in a life-threatening systemic inflammatory response syndrome, also known as “cytokine storm”. **(D)** Selected cytokine/chemokine modulation drugs that have been applied in COVID-19 clinical trials are shown in the upper right of the figure. They include neutralized antibodies (siltuximab, secukinumab, certolizumab); receptor antagonists (anakinra, sarilumab, tocilizumab), human recombinant proteins (IFN-β-1α, IFN-α-1β, IFN-α-2β); small-molecule drugs (baricitinib, remdesivir, molnupiravir, etanercept); and cell therapy (NKG2D-ACE2 CAR-NK cells). The drugs have been applied to COVID-19 patients to boost their immune systems and inhibit viral replication or cytokine storms. Figure created with BiorRender.com.

This section aims to investigate the distinct cytokine profiles associated with COVID-19 immunopathogenesis. They include interleukins (IL), interferon (IFN), tumor necrosis factor (TNF)-α, and chemokines. Changes in immune mechanisms, cytokine, and chemokine secretion caused by SARS-CoV-2 infection, and reported druggable targets are described in the following sections and summarized in **Figure 1** and **Table 1**.

**Table 1:** Representative of Histopathological Changes, Cytokine and Chemokine Secretions, and Cellular Origins in SARS-CoV-2 Infection.

Modulator	Main Cell Source	Type and Function	Clinical Feature		Reference
**Cytokines**					
IL-6	Macrophage, monocyte, activated T cell, dendritic cell, Th2, epithelial cell, endothelium cell, fibroblast	Proinflammatory cytokine; pyrogenic and antibody-enhancing function; induces acute-phase reactants Key player in COVID-19 pathology (neutrophils’ chemotaxis, lymphocytes exhaustion, and lymphocyte necrosis) and its exacerbated inflammatory responses; serum circulation positively correlates with disease severity; negative regulate T cell by increasing the exhaustion markers; main player in cytokine storms	Body	Fever, anemia, vascular leakage, interstitial edema, myocardial dysfunction, cardiomyopathy, complement & coagulation cascade activation, diffused intravascular coagulation; shock, respiratory failure, multiorgan dysfunction	(4, 6, 8, 9, 20, 22–24, 28, 36–39, 58, 65–88)
			Lung	Acute respiratory distress syndrome (ARDS), endothelial dysfunction, thrombin formation, and impaired fibrinolysis	
			Kidney	Acute kidney injury	
			Liver	Increased acute-phase protein	
IL-1β	Macrophage, monocyte, endothelium cell, activated T cell, dendritic cell	Inflammasome-induced cytokine May correlate with inflammation intensity and Th1 cell activation; activates and increases IL-6 production; dysfunction drives macrophage activation syndrome	Body	Fever	(4, 6, 8, 9, 12, 28, 67, 68, 77, 78, 89–92)
			Lung	ARDS	
			Liver	Increased acute-phase protein	
IL-18	Th1, endothelium cell, fibroblast, activated T cell, macrophage	Proinflammatory cytokine; regulates both Th1 and Th2 responses Associates with type I interferon in COVID-19; serum concentration correlates with increase in pneumonia severity	Lung	ARDS	(8, 12, 68, 93, 94)
			Liver	Liver damage	
IL-10	Macrophage, monocyte, Th1 and Th2	Anti-inflammatory cytokine; inhibits Th1 cells and cytokine release Associated with COVID-19 severity; negative regulation of T cells by an increase of exhaustion markers; may expand cytotoxic effector CD8^+^ T cells, causes hyperactivation of adaptive immunity; high concentrations correlate with low viral load.	Lung	ARDS	(70–72, 77, 79–81, 95–99)
			Liver	Increased acute-phase protein	
IL-17	Macrophage, iNKT cell, Th17, neutrophil	Proinflammatory cytokine; neutrophilic inflammation-promoting cytokine Associates with Th17 responses, viral load, severity of disease in COVID-19	Lung	Acute lung injury, ARDS	(37, 79, 98, 100–102)
IFN-γ	Macrophage, Th1 cell, Th17 cell, CD8^+^ T cell, CD4^+^ T cell, dendritic cell, NK cell	Proinflammatory cytokine; activates macrophages Impaired activity in severe disease; increased levels strongly correlate with Th1 cell activation	Body	Fever, impaired hematopoietic function, disseminated intravascular coagulation, decreased serum protein, headaches, chills, fatigue, malaise, cardiomyopathy, vascular leakage, production of acute-phase protein.	(4, 9, 12, 28, 36, 68–70, 72, 76, 77, 79, 101, 103–106)
			Lung	ARDS, lung injury	
TNF-α	Macrophage, monocyte, Th1, Th17, CD8^+^ T cell, dendritic cell, epithelial cell, endothelium cell	Pyrogenic cytokine; increases vascular permeability Impaired activity in severe patients with COVID-19; increases IL-6 production; main contributor to cytokine storm interplay; negative regulate T cell by increasing the exhaustion markers	Body	Impaired hematopoietic function, disseminated intravascular coagulation, debilitating, hyperlipidemia, flu-like symptoms	(4, 6, 8, 9, 28, 36, 37, 66, 68–72, 77–80, 86, 91, 98, 101, 105, 106)
			Lung	Alveolar edema, proteinaceous exudates, desquamation of pneumocytes, ARDS	
			Liver	Liver damage	
**Chemokines**					
CCLX	Macrophage, monocyte, activated T cell, dendritic cell, alveolar epithelial cell	CXCL9, CXCL10, CXCL11, CCL2, CCL3, CCL4, CCL7, CCL8, CCL20, CXCL6, CCL5, and IL-8 are upregulated in COVID-19	Lung	ARDS	(4, 6, 8, 9, 68, 77, 96, 107)
CXCL10	Macrophage, dendritic cell, Th2 cell	Interferon γ-inducible chemokine; recruitment of macrophages, Th1 cells, NK cells Positively correlates with Murray score, viral load, and disease severity; increased levels strongly indicate Th1 cell activation	Lung	ARDS	(9, 68, 70, 71, 77, 79, 91, 98, 108)
IL-8	Macrophage, monocyte, epithelial cell, endothelium cell	Recruits neutrophils Associates with severity of disease in COVID-19	Lung	ARDS	(4, 9, 37, 77, 79, 91, 109)
GM-CSF	Macrophage, monocyte, activated T cell, epithelial cell	Proinflammatory cytokine, stimulating proinflammatory cytokines and chemokines Associated with Th17 responses; GM-CSF secretion of immune cells (CD4^+^ T cells, CD8^+^ T cells, NK cells, and B cells) significantly higher	Lung	ARDS	(4, 6, 9, 36, 70, 77–79, 96, 110–112)
			Liver	Increased acute-phase protein	

### Interleukins (ILs)

**ILs,** a family of cytokines, are involved in immune cell differentiation and activation, directing them to the infection sites, enhancing acute-phase signaling, activating epithelial cells, and mediating secondary cytokine production (113). Both IL-6 and IL-1β are major proinflammatory cytokines released during viral infections (65) to induce acute-phase protein secretion by hepatocytes which activate the complement system. The complement system cascade further increases inflammatory and opsonization. IL-1β may also enhance inflammatory responses in the bronchi and alveoli of patients with lung injury (66, 114). Furthermore, higher IL-2, IL-17, and IL-8 levels have also been noted in patients with COVID-19 (67, 115). Of these cytokines, IL-6 deserves a more extensive discussion with respect to its involvement in the coronavirus-induced cytokine storm (68). However, a previous study showed that compared to the standard care, treatment with IL-6 and IL-1 inhibitors could reduce the neutralizing activity of SARS-CoV-2 antibodies at day 30, which may indicate that patients with cytokine-inhibitor treatments may be at risk for re-infection (116).

#### IL‐6

IL-6 is critically involved in inflammation owing to its role in regulating the acute-phase protein response (113). It is produced by nearly all stromal cells, B lymphocytes, T lymphocytes, macrophages, monocytes, DCs, mast cells, and other nonlymphocytic cells, such as fibroblasts, endothelial cells, keratinocytes, glomerular mesangial cells, and tumor cells (117). Of note, IL-1β and TNF-α increase IL-6 production (68), and in turn, IL-6 can promote T cell proliferation and resist T cell apoptosis by activating STAT3 (118). Furthermore, TGF-β, along with IL-6 and IL-21, promotes Th17 cell development by enhancing RORγt expression (119). IL-6 can also activate Th2 cytokines and dampen the production of T_Reg_ cells via the STAT3 pathway (120–124). Moreover, high IL-6 levels can activate the coagulation system and increase vascular permeability, thereby enabling the rapid spread of inflammation (69). IL-6 dysregulation has also been described to promote cell necrosis and apoptosis (125). Owing to these pleiotropic properties, IL-6 plays an important role in the pathogenesis of the cytokine storm (68).

Recent studies have suggested that the spleen and lymph nodes are infiltrated by macrophages that express ACE2 and that the nucleoprotein antigen is significantly associated with IL-6 production. This suggests that they contribute to excessive inflammation in COVID-19 (6, 70, 107, 126). Further, IL-6 produced by CD14^+^CD16^+^ monocytes was significantly higher in ICU-treated patients than in non-ICU patients (6, 127). SARS-CoV-2 can dysregulate host immune responses. For example, the number of Th17 cells could be raised by a virus-driven increase in IL-6 production (68). SARS-CoV-2 may also elevate the levels of IL-10, and TNF-α, which would negatively regulate T cells by increasing exhaustion markers such as PD-1 or Tim-3 (71, 72). Moreover, IL-6 plays a crucial role in the pathology of COVID-19 as it modulates the chemotaxis of neutrophils and lymphocyte exhaustion and necrosis (38). Patients with COVID-19 also display selective induction of the macrophages that produce IL-6, but not TNF-α and IL-1β, and this then directly promotes lymphocyte necrosis (38).

Systematic reviews and meta‐analyses have found higher serum levels of IL-6 in >50% of all patients with COVID-19 (70, 128). Moreover, patients who subsequently developed certain adverse clinical outcomes, including the cytokine storm, ICU admission, acute respiratory distress syndrome (ARDS), or death tended to have higher IL-6 levels (5, 70, 89, 129) than healthy cohorts. The IL-6 levels were elevated nearly threefold in complicated COVID-19 cases, and there were several systemic and extrapulmonary disorders not found in patients with uncomplicated disease (28, 38, 39, 67, 68, 73, 103, 130–133).

In addition to the level association, a combination of multiple cytokines, including IL-6, IL-10, and IL-8, combined into one score may also predict disease severity (74). Thus, serum IL-6 levels (>24 pg/mL) can be used at the initial assessment to predict hypoxemia that requires hospitalization. This may help in the early identification of patients who need hospitalization because it has excellent sensitivity and good specificity (134, 135).

As they do in SARS-CoV, high serum IL-6 levels correlate with shock, respiratory failure, ARDS, and multiorgan dysfunction in severe COVID-19 (58, 73, 75). IL-6 overexpression can induce other complications, including fever, vascular leakage, anemia, cardiomyopathy, acute kidney injury (AKI), interstitial edema, and myocardial dysfunction. In support of this, patients with severe COVID-19 (5) exhibited higher IL-6/IFN-γ ratio and greater lunch damage while a higher risk of hypercytokinemic immune dysregulation (or cytokine syndrome), respiratory failure, and death (76) was characterized by elevated IL-6 levels ≥80 pg/mL. When assessed in parallel, the findings could be associated with a cytokine storm favoring lung damage, than those with moderate COVID-19 (104). Taken together, blocking the IL-6 signaling pathway might inhibit excessive inflammation from SARS-CoV-2 infection (136).

To evaluate the therapeutic efficacy of IL-6 inhibition, clinical trials have tested several monoclonal antibodies, including siltuximab (IL-6 neutralization), sarilumab (IL-6 receptor antagonist), and tocilizumab (IL-6 receptor antagonist) (137). The earliest IL-6-blocking therapies first emerged in China (138), and most of the randomized trials with IL-6 receptor blockers did not have a significant effect on mortality (139–143). Tocilizumab showed no significant improvement in clinical outcome or mortality compared with placebo (140). Nevertheless, recent studies that used a combination of tocilizumab and siltuximab have shown promising results, including clinical and radiographical improvements, as reported by the Randomized, Embedded, Multifactorial Adaptive Platform Trial for Community-Acquired Pneumonia (REMAP-CAP) (144) and other studies (144–147). In the REMAP-CAP study, mortality among patients treated with tocilizumab and sarilumab was 27% compared with 36% in the control group (144). Furthermore, one of the largest initial observational studies in New Jersey has shown reduced mortality owing to tocilizumab, in particular, the mortality rate among 547 ICU patients with COVID-19 treated with tocilizumab was 46% compared with 56% among 134 individuals provided standard care (147). At present, the therapeutic efficacy of IL-6 inhibitors in patients with COVID‐19 appears promising; however, more evidence is needed to establish a definitive benefit (73).

#### IL-1 Family

Several well-known cytokines in the IL-1 family, including IL-1β and IL-18, have important roles in inflammation, hematopoiesis, and fibrosis (148), and SARS-CoV-2 has been shown to induce the secretion of IL-1 family cytokines, where their levels correlate with virulence (5, 130, 149). Some reports have also indicated that higher levels of IL-1 family cytokines present in the plasma of patients with COVID-19, that levels are higher in severe cases compared with mild cases, and that the levels strongly associate with the Murray score (57, 108, 130, 149, 150). Of note, gene expression analyses concur with these findings, i.e., IL-1-related proinflammatory pathways are highly upregulated in severe cases (151). Therefore, the IL-1 blockade treatment by anakinra (an anti-IL-1 receptor antagonist) may be an effective alternative option for COVID-19 patients (152).

##### IL-1β

IL-1β is a proinflammatory cytokine that not only responds to infection and inflammation but also plays an important role in acute and chronic autoinflammatory diseases (153). Elevated IL-1β levels have been associated with SARS, hypercoagulation, disseminated intravascular coagulation, and most severe COVID-19 cases (100, 154). Clinical and laboratory parameters indicated that SARS-CoV-2 appears to promote both the activation and maturation of IL-1β and this, in turn, activates other proinflammatory cytokines, including IL-6 and TNF-α. Further, IL-1β dysfunction drives Th1 cell activation (12, 68), macrophage activation syndrome (8, 28, 67, 89, 90), fever (77), ARDS (8, 77, 78), and cytokine storm (100, 155). Using single-cell transcriptomic (156, 157) and flow cytometric analyses (90, 158), some studies have identified that peripheral blood mononuclear cells (PBMCs) from patients with COVID-19 contained IL-1β-associated inflammasome signatures (159).

#### IL-18

IL-18 is a proinflammatory cytokine produced by macrophages during the very early stages of a viral infection (160). It induces the production of IL-6 and IFN-γ, which are considered important for optimal viral host defense (160, 161). It also drives Th2 cytokine production from CD4^+^ T cells and NK cells and regulates both Th1 and Th2 responses (93). Additionally, IL-18, along with other cytokines (CCL2, CXCL10, CCL20, IL-18, IL-3, IL-6, G-CSF, GM-CSF, IFN-γ), is suggested to be involved in regulating neutrophil function (162). The activated neutrophils contributed to the formation of neutrophil extracellular traps (163) and increased neutrophil-induced inflammatory response (162). In addition, aberrant production of IL-18 can cause severe tissue injury (160). Patients with severe COVID-19 had higher IL-18 serum concentrations than those with mild symptoms (94, 160), with no difference in IL-18 concentrations between healthy subjects and asymptomatic COVID-19-infected individuals (160). As serum IL-18 concentrations consistently rise with an increase in the severity of pneumonia, significantly higher concentrations were observed in patients who developed macrophage activation syndrome, ARDS (164), or liver damage (77). Further, serum IL-18 levels were higher in males than in females (94, 164). Thus, the serum IL-18 concentration may be used to identify patients who might subsequently need hospitalization (160).

#### IL-10

IL-10 functions as an anti-inflammatory cytokine, and it is a crucial feedback regulator that exerts immunosuppressive effects on both innate and adaptive inflammation, autoimmune pathologies, and Th1 cell activity (79, 95, 165–173). It is secreted by multiple cell types, including Th1, Th2, Th17 cell subsets, T_Reg_ cells, CD8^+^ T cells, B cells (167, 174–178), mast cells, eosinophils, macrophages, and DCs (179–181). IL-10 is dramatically elevated in COVID-19, and this was believed to be a negative feedback mechanism to suppress inflammation and to induce ARDS (96), acute-phase protein response (77, 97), severe pneumonia, and damage to vital organs (33, 71, 107, 182, 183). Studies have reported higher IL-10 levels in ICU patients than in non-ICU patients (33, 71, 95). In addition, the increased number of IL-10-producing regulatory T cells could be correlated with long-term viral persistence (184). Hence, several lines of clinical evidence suggest that such dramatic and early elevation of IL-10 might exacerbate pathogenesis in determining COVID-19 severity (95). Moreover, the increased IL-10 expression is considered an indicator of poor prognosis in COVID-19 (97, 183). In contrast, other recent studies have demonstrated that IL-10 may directly expand cytotoxic effector CD8^+^ T cells and contribute to the hyperactivation of adaptive immunity in patients with COVID-19 (95). This observation lent credibility to the idea that both anti-inflammatory and immune-activating roles of IL-10 may occur simultaneously in COVID-19 (68, 95, 185).

#### IL-22

IL-22 belongs to the IL-10 cytokine family and is produced by γδ T cells, Th1 cells, Th17 cells, Th22 cells, NKT cells, and group 3 innate lymphoid cells (ILC3) (186–188). In general, IL-22 is a pro-and anti-inflammatory cytokine involved in tissue inflammation, immunosurveillance, tissue repair, and homeostasis through the IL-22R activation pathway (186, 189–195). In patients with SARS-CoV-2 infection, IL-22 plasma levels were correlated with disease severity (12, 196), and they were positively correlated with the Th17 population. The increased Th17 proportions and IL-22 were observed in cases with headache, hyperinflammation, respiratory system dysfunction, and ARDS (163, 197). Other studies indicated that IL-22 levels are negatively correlated with the Th22 levels (188, 198). Functional assays revealed that both peripheral blood mononuclear cells (PBMCs) and a human bronchial epithelial cell line (16HBE) can produce IL-22 (188). This suggests that IL-22 could be expressed differently in peripheral blood and tissues.

#### IL-17

IL-17 is produced by Th17 lymphocytes and other cells, including CD8^+^ cells and γδT cells, NK cells, and ILC3 (199). IL-17 acts as a neutrophilic inflammation-promoting cytokine (79) whose levels increase during inflammatory processes and autoimmune diseases (200–203). It also plays a role in tissue damage, physiological stress, and infection (100). IL-17, along with IL-22 and TNF-α, induces the production of antimicrobial peptides in the gastrointestinal tract and skin (204, 205). In addition, because both IL-17 and IL-22 originate in Th17 cells, the dysregulation of the above cytokines could contribute to several proinflammatory diseases (206). Therefore, applying Th17-inhibiting therapies such as thiamine to reduce the Th17 mediated pro-inflammatory response (101) may be an effective treatment for decreasing cytokine storms (207). In one study, 16 patients a proinflammatory state from alcohol use disorder were treated with thiamine, and it significantly reduced IL-17 levels and ameliorated the Th17 responses (207).

Upon SARS-CoV-2 infection, IL-17 levels, along with other Th17 related proinflammatory cytokines, such as IL-1, IL-6, IL-15, TNF, and IFN-γ, are increased, which has a positive correlation with disease severity (101, 102, 208). Previous reports indicating the elevation of IL-17 levels in patients with SARS-CoV or MERS (209, 210) as well as elevated IL-17 levels in SARS-CoV-2-induced cytokine storm (33), ALI (102), ARDS (37), viral load have been reported (77, 211). A Janus kinase 2 inhibitor, Fedratinib, has been used to decrease IL-17 expression by inhibiting Th17 cells in murine models (212). By contrast, a recent study illustrated that IL-17 levels were not significantly different between uninfected individuals and patients with COVID-19 with severe versus mild symptoms (115). In addition, secukinumab, a human anti-IL-17 neutralizing monoclonal antibody, also showed no significant difference in adverse effects in COVID-19 patients (43).

#### IL-33

IL-33 is an alarming and crucial immune modulator released by endothelial and epithelial cells, activated fibroblasts, fibroblast-like cells, and myofibroblasts to maintain the tissue and immune homeostasis and inflammation through IL-33/ST2 signaling (211, 213). IL-33 plays important roles during allergic, fibrotic, infectious, and chronic inflammatory diseases by activating the different immune subgroups, such as Th1, Th2, T_Reg_, CD8^+^ T cells, mast cells, group 2 innate lymphoid cells (ILC2s), granulocytes, macrophages, DCs, NK cells, iNKT cells, and B cells (209, 210, 213, 214).

In COVID-19 patients, increased IL-33 serum levels were associated with poor outcomes (209, 215–217). IL-33 activation could induce a type-2 immune response by inducing ILC2s (217). These might help differentiate pathogenic γδ T cells (216), thus contributing to pulmonary fibrosis induced by viral infection (216, 218). In addition, transcriptomic analysis of bronchoalveolar lavage fluid (BALF) from COVID-19 patients showed an increased population of IL-33-producing cells with the disease severity (219) and strong upregulation of IL-33 compared to healthy samples (220).

### Interferons (IFNs)

IFNs encompass a family of cytokines that play a central role in providing efficient protection against viral infections by activating the antiviral or immunomodulatory properties (221, 222). Human IFNs have been classified into three major types based on signal receptors: IFN type I (IFN-I; IFN-α and IFN-β), IFN type II (IFN-γ), and IFN type III (IFN-λ1) (223). Type I IFNs response can be rapidly triggered when host cells recognize PAMPs such as viral nucleic acids and have been known to combat viruses by inducing the expression of IFN-stimulated genes (ISGs) in epithelial cells, which exert antiviral functions, inhibit viral replication, and indirectly stimulate both innate and adaptive immune responses (3, 68, 224, 225).

While IFN-β-1α and IFN-β recombinant protein have shown great potential in inhibiting SARS-CoV-2 replication *in vitro* (226). Paralleling the potential, IFN-λ 1/3 has also been induced in early phases of infection and in convalescent COVID-19 patients (227). The results indicated that IFN-β and IFN-λ1 could block virus infection and inhibit the production of SARS-CoV-2 (226, 228). In addition, the dysregulated production of type I IFNs and the exacerbated release of proinflammatory cytokines makes COVID-19 more severe (226, 229). At the same time, the reduced type II IFN was correlated with disease severity *in vitro* (230), and the IFN-γ plasma levels of COVID-19 ICU patients have been significantly reduced compared to other cohorts (227).

It has been noted that type I IFN, especially IFN-β, can be effective against SARS-CoV-2 to tackle severe COVID-19 and prevent clinical deterioration. Analogously, SARS-CoV-2 may also be sensitive to IFN (231, 232). To evaluate the therapeutic efficacy of IFN-β, several studies in REMAP-CAP and the WHO’s Solidarity Trial administered IFN-β recombinant protein as a potential therapy against COVID-19 (45, 231, 232). A combination of IFN-β-1α, lopinavir/ritonavir, and ribavirin could eliminate the virus from the nasopharyngeal swabs in a phase II clinical trial (45, 233). Another study has shown IFN-β-1α combined with lopinavir/ritonavir or atazanavir/ritonavir and hydroxychloroquine significantly decreased mortality on day 28 in a cohort of 42 severe COVID-19 cases (234). In addition to the combination, pegylated interferon alfa-2β (PEG IFN-α-2β) along with the standard of care (SOC) enabled a faster viral reduction in patients with moderate COVID-19 than with standard care alone (235). The pegylated interferon lambda (PEG IFN-λ) treatment particularly in patients with a high baseline viral load (80), accelerated viral decline and increased the proportion of patients with viral clearance by day 7 has shown a similar role as PEG IFN-α-2β.

#### IFN-I

IFN-I can be produced by many cell types, including DCs, lymphocytes, macrophages, fibroblasts, endothelial cells, and osteoblasts (236–238). IFN-I can further stimulate both macrophages and NK cells *via* IRF3/IRF7 antiviral signaling to counter viral infections, and in response to PAMPs, plasmacytoid DCs have been identified as the most potent and natural type I IFN-producing cells (229). Nevertheless, viruses can counteract IFNs by harnessing both structural and nonstructural proteins and can also efficiently suppress IFN induction (239). Of note, such inefficient IFN response may account for progressive virus replication, cytokine storm development, and death in SARS (240).

Many viruses, including SARS-CoV-2, have evolved mechanisms to evade the antiviral effects of IFN, and patients with COVID-19 tend to have suppressed responses to IFN (type I, II, or III) along with impaired monocytes, macrophages, and neutrophils (35, 72, 80). Compared with other respiratory RNA viruses, SARS-CoV-2 is also a poor inducer of IFN-I, both *in vitro* and in animal models (35, 241). Patients without IFN-α production had poorer outcomes and a higher viral load. Thus, screening patients for IFN production after COVID-19 diagnosis could be crucial in selecting those who could benefit from early intervention with IFN treatment (242). Recent studies suggest that an impaired response of IFN-I in the early stage of the disease may lead to an acute phase and play a major role in creating the cytokine storm (243). Adding to the study, a recent working hypothesis has suggested that the timing of IFN response to SARS-CoV-2 infection varies based on viral load and genetic differences (244) with low viral loads characterized by an IFN response suitable for viral clearance, resulting in mild infection. In contrast, a high viral load can delay IFN response and consequently lead to severe disease (245). Studies have suggested that patients with mild or moderate COVID-19 exhibit greater type I IFN response during days 8-12 compared with patients with severe disease (4, 8). Moreover, some infected patients expressed a low level of IFN-I, but ISGs expression was enhanced in patients’ BALF (240). This suggests that even limited IFN-I production is sufficient to express relevant genes (72, 246).

Despite early reports to the contrary, increasing evidence has highlighted a role for IFN-I responses in severe COVID-19 development (94). A robust type I IFN response has been reported in patients with severe COVID-19, which through diverse mechanisms, exacerbated hyperinflammation (94, 247). Further, a recent longitudinal analysis illustrated that IFN-α levels in peripheral blood remained high in patients with severe COVID-19 and that classical monocytes exhibit both IFN-I and TNF/IL-1β-driven inflammatory responses (94), and approximately 10% of patients with severe COVID-19 had neutralizing immunoglobulin (Ig)G auto-antibodies against type I IFNs (248, 249); hence these patients required plasma exchange (250). Of note, contradictory results among multiple studies on IFN-I responses in patients with COVID-19 might be explained by differences in the definition of disease severity, sampling time points, and/or readout type (for example, IFN-I itself or cellular responses to IFN-I) (247).

### TNF-α

**TNF-α** is a pyrogenic cytokine secreted by macrophages, monocytes, Th1 cells, Th17 cells, CD8^+^ T cells, and DCs during the acute phase of inflammation or infection (68, 77, 79). It is a central cytokine in viral diseases that increases vascular permeability (251) and is associated with several chronic inflammatory conditions and autoimmune diseases (68). For >20 years now, anti-TNF antibodies have been used for alleviating the severity of some autoimmune inflammatory diseases, including rheumatoid arthritis, inflammatory bowel disease, and ankylosing spondylitis. Further, the United States Food and Drug Administration has approved four off-label indications for anti-TNF therapy, implying that TNF is a valid target in multiple inflammatory diseases (244, 252).

COVID-19 is characterized by unique hyperinflammatory signatures across all types of immune cells, particularly the upregulation of TNF-α**-**, IL-6-, and IL-1-driven inflammatory responses in severe disease (35). TNF-α presents in the blood and lungs of patients with COVID-19 (244), and in severe COVID-19 cases, high systemic TNF-α levels were associated with respiratory distress syndrome (98, 245, 253, 254), and lower survival along with pulmonary dysfunction [edema, proteinaceous exudates, pneumocyte desquamation, and ARDS (66)] as well as impaired hematopoietic function, disseminated intravascular coagulation, debilitating hyperlipidemia, liver damage, chronic kidney disease, diabetes, and hypertension (8, 33, 77, 135, 255). Thus, high TNF-α levels can be an independent predictor of patient survival (135).

As TNF-α overproduction has been documented in COVID-19, the efficacy and clinical benefits of anti-TNF antibody therapy have been investigated (244). One clinical trial showed that etanercept, a TNF-α inhibitor, can attenuate disease in patients with severe COVID-19 by suppressing their systemic autoinflammatory responses (256), and patients with COVID-19 previously prescribed etanercept treatment did not develop severe COVID-19 (257). Likewise, another anti-TNF-α antibody, certolizumab, may have beneficial effects in patients with COVID-19 (100, 258). A recent study showed that inhibiting both TNF-α and IFN-γ in multiple cytokine storm models protected against death in SARS-CoV-2 infection and other inflammatory syndromes, such as sepsis, hemophagocytic lymphohistiocytosis, and cytokine shock (259). It is still not clear whether a single cytokine blockade (such as TNF-α inhibitors) could be effective in CRS associated with COVID-19. Nevertheless, some results show that anti-TNF therapy should be explored on hospital admission as it may prevent the need for intensive care (244).

Histopathological changes and cytokine and chemokine secretions along with cellular origins in SARS-CoV-2 infection, which are described in the preceding and following sections, are summarized in **Table 1**.

## Chemokines Induced by SARS-CoV-2 Infection

Chemokines, a family of small cytokines, are the crucial mediators of appropriate immune responses (260). However, their excessive release is the primary cause of hyperinflammation, which may be a direct cause of ARDS (261). The exaggerated chemokine response is critical in several viral diseases, such as SARS, MERS, influenza, and SARS-CoV-2 (262). Further, several clinical investigations have revealed that chemokines such as IL-8, CCL2, CCL3, CCL7, CCL8, CXCL2, CXCL16, and CX3CL1 are infiltration signals that mediate the recruitment of mononuclear phagocytes to lungs (6, 240, 261, 263), and they are directly involved in the pathogenesis of severe clinical sequelae in COVID-19, including major complications that cause death in approximately 40% of severe COVID-19 cases (66, 68, 77, 261, 264). We review the role of chemokines in COVID-19 pathogenesis to improve the understanding of the disease immunopathology, which may further aid in developing possible therapeutic targets for COVID-19 (261). Chemokine secretions and their cellular origins in SARS-CoV-2 infection are summarized in **Figure 1**, **Table 1**, and described in the following sections.

### CXCL10/IP-10

CXCL10, also known as IP-10 or small-inducible cytokine B10, belongs to the C-X-C chemokine family. It binds to the CXCR3 receptor and acts as a chemoattractant for immune cells. That enhances chemotaxis, apoptosis, cell growth, angiostasis, and recruitment of macrophages, Th1 cells, and NK cells (265). Alterations in CXCL10 expression have been associated with inflammatory diseases (including infections), immune dysfunction, and the development of tumors. Thus, CXCL10 is a recognized biomarker of varying progression and severity of disease (265), so it might be used to detect early stages of SARS (68). In COVID-19 cases, levels of CXCL10 serum are highly associated with disease severity, viral load (68, 108), and the Murray score (71, 108). CXCL10 has been found in the BALF from patients with COVID-19 who had higher proinflammatory genes (220). In addition, higher circulation levels of CXCL10 can activate Th1 cell function, and it has been shown to be highly correlated with COVID-19-induced ARDS in clinical and experimental studies (266). Therefore, CXCL10 may also be considered a prognostic and potential therapeutic marker for COVID-19 progression (108).

### IL-8/CXCL8

IL-8 or CXCL8 is produced by macrophages and other cell types, such as epithelial cells, airway smooth muscle cells, and endothelial cells (135, 267, 268). Elevated serum IL-8 levels have been observed in many diseases, including severe lung injury, and in patients who developed adverse outcomes in SARS-CoV and MERS-CoV infections (269–272). Upon SARS-CoV-2 infection, increased IL-8 concentrations are associated with ARDS (77) and COVID-19 severity (87, 135, 255, 268, 273–275). Irrespective of demographics or comorbidities (106, 135, 276, 277), lower IL-8 was associated with a shorter duration of illness and in convalescent, recovered, and asymptomatic (276, 278). As a neutrophil chemotactic factor, IL-8 recruits neutrophils to the infection site and activates them (135, 279). Therefore, neutrophilic infiltration, neutrophil-lymphocyte ratio, and increased IL-8 expression are considered realistic prognostic biomarkers of COVID-19 progression and severity (10, 30, 135, 280, 281). However, the correlation between IL-8 levels and disease severity remains controversial (135, 273–275, 281, 282) as the positive correlation is shown only in univariate and not in multivariate analysis (88). Interestingly, the correlation between sex and IL-8 is also unclear in that while some studies have documented no significant differences in serum IL-8 levels (135), others have reported lower IL-8 expression in female SARS-CoV-2 patients than in male patients (275).

### GM-CSF

GM-CSF, a proinflammatory cytokine and chemokine also known as CSF2, is a member of the CSF superfamily (112). This monomeric glycoprotein has several cellular origins, including macrophages, T cells, mast cells, NK cells, endothelial cells, and fibroblasts (4, 9, 36). GM-CSF was initially classified as a hematopoietic growth factor, yet it is now believed to play an essential role in communicating between tissue-invading lymphocytes and myeloid cells by stimulating the secretion of proinflammatory cytokines and chemokines (such as IL-1, IL-6, and TNF) (112). Compared with healthy controls, the percentage of GM-CSF-secreting immune cells, such as CD4^+^ T cells, CD8^+^ T cells, NK cells, and B cells, was significantly higher in patients with COVID-19 (110, 111). Increased GM-CSF can induce greater acute-phase protein expression (77) and activate Th17 responses (98) to contribute to COVID-19 pathogenesis (33). In addition, to the COVID-19 patients, particularly those admitted in ICU, pathogenic Th1 cells increased rapidly along with GM-CSF and IL-6 secretion (49, 111, 283). Such a proinflammatory environment can further trigger CD14^+^CD16^+^ monocytes to secrete more GM-CSF and IL-6. These aberrant and numerous GM-CSF^+^-IL-6^+^ cells may enter the lungs and create a hyperinflammatory environment, thereby worsening the cytokine storm in patients with COVID-19 (10, 49, 111). Thus IL-6 and GM-CSF are also considered markers of poor prognosis in the later stages of COVID-19, and these proinflammatory mediators can predispose patients to respiratory failure and eventually ARDS (49).

In current COVID-19 clinical trials, the administration and inhibition of GM-CSF are being therapeutically tested. The inhibition of GM-CSF signaling may function as a potential therapeutic target in patients with COVID-19-associated hyper inflammation and ARDS (49, 284). At present, there are multiple trials with anti-GM-CSF drugs (e.g., Otilimab, Lenzilumab, Namilumab, Gimsilumab, and TJ003234) or GM-CSF receptor antagonists (e.g., Mavrilimumab) (110, 276). On the other hand, the FDA-approved recombinant GM-CSF, Sargramostim (Leukine^®^), is currently being investigated in a phase IV trial as an adjuvant therapy to restore alveolar anti-inflammatory macrophages to manage acute hypoxic respiratory failure and ARDS (NCT04326920) (57, 110, 276).

### CCL2/MCP-1

CCL2, also known as monocyte chemoattractant protein-1 (MCP-1), is produced by many cell types, including endothelial cells, fibroblasts, epithelial cells, smooth muscle cells, mesangial cells, astrocytes, monocytes, and microglia (277, 278, 285–287), serving as a regulator of monocyte/macrophage migration and infiltration. The migration of monocytes through the bloodstream to the vascular endothelium plays a key role in the response to inflammation (286, 288, 289). Both CCL2 and its receptor, CCR2, are known to be activated and involved in several types of inflammatory diseases, such as Alzheimer’s disease, parkinsonism, stroke, ischemic heart disease, arthritis, and COVID-19 (286, 289). With COVID-19, the increased CCL2 is not only associated with respiratory failure but also with extrapulmonary manifestations. The increased CCL2, along with IL-1, IL-6, TNF-α, MMP-8, and ICAM-1, can increase the permeability of the barrier between blood and cerebrospinal fluid, increasing the inflammatory infiltration (290–292). In combination with other inflammatory cytokines, elevated CCL2 can also increase the severity of neurodegeneration, cognitive dysfunction, and stroke (293). Moreover, increased CCL2 levels were reported to be correlated with the development of acute kidney injury in critically ill COVID-19 patients (294), and patients with higher CCL2 expression tend to have detrimental disease progression.

### CX3CL1

CX3CL1, also known as fractalkine (FKN), is highly expressed by activated endothelial cells and is known to be involved in endothelial dysfunction, contributing to the development of atherosclerosis and other cardiovascular events (295–297). Additionally, CX3CL1 serves as an infiltration signal that mediates mononuclear phagocyte recruitment to the lungs (261). In COVID-19 patients, increased CX3CL1 is associated with disease severity (298), and patients with neurological syndrome (NS) presented with higher CX3CL1 levels than non-NS patients (299). Studies have shown that CX3CL1 might promote neurological vascular damage and thrombosis during SARS-CoV-2 infection. Spike protein-mediated endocytosis reduces AEC2 in the cell membrane, upregulating the expression of CX3CL1 in the endothelium (300). Increased CX3CL1 can promote a pro-thrombotic environment and enhance immune cell recruitment, leading to more severe COVID-19 and mortality (301). Thus, CX3CL1 may serve as a predictive marker for identifying COVID-19 patients who are at risk of developing thrombotic complications and require more aggressive anti-thrombotic management (301).

## Clinical Symptoms

The incubation period of COVID-19 ranges between 1 and 14 days. However, the period is predominantly 3-7 days (302–304). Immune response to SARS-CoV-2 involves both innate and adaptive immunity (3), and although differentiated lymphocytes can take days or weeks to become functional, they have an important role in controlling and shaping host immune responses as they provide multiple immune-related functions and long-lasting protection. In particular, while T cells can either kill an infected cell *via* cytotoxic CD8^+^ T cells or balance immune response with the help of CD4^+^ helper T cells, B cells produce antibodies against pathogenic antigens, which is also known as humoral immunity (305). Both innate and adaptive immune cells are critical for eliminating SARS-CoV-2 infection as they orchestrate the direct clearance and eradication of pathogens and contribute to the generation of long-term adaptive immune responses (10, 80, 306). The hyperactivity of the immune system stimulates the production of several cytokines, including IL-6, IL-8, IL-1β, IL-2, IL-4, IL-7, IL-10, IFN-γ, TNF-α, GM-CSF, CCL2, CCL3, CCL5, and CXCL10 (100) (as described in the previous sections). Cytokine- or chemokine-specific clinical symptoms are as follows: elevated IFN-γ causes fever, headaches, chills, fatigue, malaise, cardiomyopathy, vascular leakage, lung injury, and acute-phase protein production; increased TNF-α causes flu-like symptoms (69, 106); and IL-6 overexpression induces cardiomyopathy and vascular leakage, and it activates the complement pathway, coagulation cascade, diffuse intravascular coagulation, and the cytokine storm (69, 82, 84, 85).

**Figure 2:**
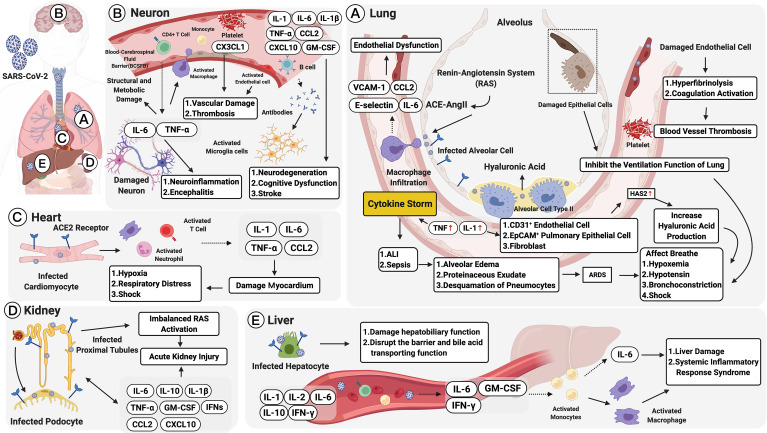
Multiple organ failure in SARS-CoV-2: pulmonary and extrapulmonary manifestations. Pulmonary manifestations: **(A)** Alveolar cells are infected by the angiotensin-converting enzyme 2 (ACE2) receptor. This leads to acute lung injury (ALI), activation of the renin-angiotensin system (RAS), and macrophage recruitment. Macrophages, activated by the ACE2-Ang II axis, can secrete IL-6, CCL2, VCAM-1, and E-selectin leading to endothelial dysfunction. Damaged endothelial cells may cause hyperfibrinolysis, activate coagulation, and increase inflammatory cytokine levels (IL-1 and TNF), which are strong inducers of hyaluronic acid-synthase-2 (HAS2) in CD31^+^ endothelium, EpCAM^+^ lung alveolar epithelial cells, and fibroblasts. The increased hyaluronic acid may cause bronchoconstriction. Extrapulmonary manifestations: **(B)** In neurons, elevated TNF-α and IL-6 secretions could increase the infiltration of immune cells and the permeability of the blood and cerebrospinal fluid barrier (BCSFB). CX3CL1 could also promote neurological vascular damage and thrombosis. IL-6 along with GM-CSF, IL-1β, TNF-α, CCL2, and CXCL10, could increase the severity of neurodegeneration, cognitive dysfunction, and stroke. **(C)** In the heart, infected cardiomyocytes would induce elevated secretions of IL-1, IL-6, TNF-α, and CCL2, damaging the myocardium and developing into hypoxia, respiratory distress, and even shock. **(D)** In the kidney, SARS-CoV-2 infects ACE2 expressing proximal tubules and the podocytes. The infected podocytes promote an imbalanced RAS, which leads to acute kidney injury (AKI). Moreover, IL-6, IL-1β, IL-10, GM-CSF, IFNs, CXCL10, CCL2 and TNF-α promote AKI persistence. **(E)** In the liver, infected hepatocytes trigger elevated serum IL-1, IL-2, IL-6, IL-10, and IFNγ, as well as pathogenic cytokine secretions mediated by T cells (GM-CSF, IL-6, and IFN-γ). The immune-mediated injury would lead to liver dysfunction and systemic inflammatory response syndrome (SIRS). Figure created with BioRender.com.

COVID-19 initially presents with “flu”-like symptoms include fever, dry cough, myalgia, fatigue, dyspnea, and anorexia. Atypical presentations are diarrhea and nausea, which can progress to life-threatening systemic inflammation like the CRS (33, 303, 304, 307, 308), which can cause multiple lung immunopathologies, including acute lung injury (ALI), systemic inflammatory response syndrome (SIRS), and ARDS (65, 129). Further, accumulating evidence demonstrates that COVID-19 has an extrapulmonary involvement; this includes the neurological, olfactory, cardiovascular, digestive, hepatobiliary, renal, endocrinological, and dermatological systems, with more than one-third of the patients exhibiting a wide range of neurological symptoms involving the central and/or peripheral nervous systems (10, 308–311). Moreover, in patients with severe COVID-19, cardiovascular complications are accompanied by the accumulation of inflammatory mononuclear cells (e.g., neutrophils) in infected vascular endothelial cells (308). Severe cases also show impaired hepatic function and a higher rate of acute kidney injury (AKI). In patients with prolonged illness, gastrointestinal symptoms also frequently occur. Endocrinological manifestations include exacerbating hyperglycemia, euglycemic ketosis, and diabetic ketoacidosis (312), and thrombosis and visceral embolization can trigger tissue hypoxia and ischemia in patients with COVID-19 (5, 313). Changes in SARS-CoV-2 infection, including pulmonary and extrapulmonary manifestations, are described in the following sections and summarized in **Figure 2**.

### Cytokine Storm Syndrome

The terms “cytokine storm” and “CRS” refer to the overproduction of inflammatory cytokines by hyperactivated immune cells. This occurs because of immune dysregulation of varying etiology, and it can cause systemic inflammatory syndromes and life-threatening multiorgan dysfunction (79). During a cytokine storm, elevated levels of circulatory cytokine drive a positive feedback loop in immune cells. This leads to the continuous recruitment of these cells to the sites of inflammation, causing an exponential increase in both inflammation and organ damage (66).

Cytokine storms are associated with various infectious and noninfectious diseases (65), including cancer, autoimmune conditions, and monogenic disorders (79). Despite the variable initial drivers of a cytokine storm, its late-stage clinical manifestations converge and often overlap. As a result, patients exhibit hyperinflammation, coagulopathy, low platelet counts, fatigue, anorexia, headache, rash, diarrhea, arthralgia, myalgia, spontaneous hemorrhage, and neuropsychiatric symptoms. These manifestations are directly caused by cytokine-induced tissue damage, acute-phase physiological changes, or immune cell-mediated responses (79). In addition, many patients develop respiratory symptoms (cough and tachypnea), which can progress to acute respiratory distress syndrome (ARDS) along with hypoxemia that may require mechanical ventilation (314). Fever, renal failure, acute liver injury or cholestasis, and occasionally, stress-related or Takotsubo-like cardiomyopathy are the characteristic manifestations of a severe cytokine storm (315).

In COVID-19, the virus stimulates various infected cells, including lung epithelial cells and alveolar macrophages, to release cytokines and chemokines. The primary cytokines involved in cytokine storms and CRS are ILs, IFNs, TNFs, CSFs, and the chemokine family, among others (100). These, in turn, activate macrophages, DCs, and other immune cells (66) to induce an aggressive inflammatory response and the subsequent release of a large number of proinflammatory cytokines to trigger a cytokine storm (28, 33, 39, 316–318). After which, chemokines recruit additional inflammatory cells, such as monocytes and phagocytes, to the inflammation site resulting in a cascading amplification of infection-induced inflammatory response (66). The abnormal and uncontrolled cytokine storm has been observed in critically ill or severe COVID-19 cases (68), and it triggers a systemic inflammatory response, leading to lung immunopathology and multiple organ failure (65, 66, 129, 319). In fact, the SARS-CoV-2-induced cytokine storm is believed to be the cause of sepsis in 28% of all fatal COVID-19 cases (320). Thus, targeting cytokines could improve survival rates and reduce mortality (11). Any potential treatments should include the direct elimination of coronaviruses, prevention strategies (e.g., vaccine development), and counteracting the imbalance and hyperactivity of the immune system.

### Pulmonary Manifestations

Histopathological changes in COVID-19 occur primarily in the lungs (304). Upon first contact with the respiratory mucosa, the virus binds to the surface receptors of ACE2 and TMPRSS2 to enter the alveolar cell (14); this event activates the renin-angiotensin system (20, 21). In the first few days after infection, innate immune cells [including macrophages, DCs, and monocytes (**Figure 2**)] recognize viral pathogen-associated molecular patterns (PAMPs) *via* PRRs (10, 15). The activated ACE2-Ang II axis can trigger macrophage infiltration and induce the secretion of several cytokines, including IL-6, CCL2, VCAM-1, and E-selectin, which induce endothelial dysfunction and, subsequently, coagulation (20, 22–24, 65). Therefore, an increase in plasma Ang II levels is considered a marker that correlates with viral load and lung injury in COVID-19 cases (10, 66). IL-1β can enhance inflammatory responses in the bronchi and alveoli in patients with lung injury, and the levels of inflammatory cytokines such as IL-1 and TNF are elevated in the lungs of patients with COVID-19. The increased levels are believed to represent a significant induction of hyaluronic acid-synthase-2 (HAS2) in the CD31^+^ endothelium, EpCAM^+^ alveolar epithelial cells, and fibroblasts, which may lead to bronchoconstriction (321).

An unbalanced immune response and elevated proinflammatory cytokines contribute to the major complications of pulmonary coronavirus infection, including sepsis, ARDS, acute lung injury (ALI) (66, 71), SIRS (65), and PF (322, 323); these complications result in respiratory failure and subsequent mortality (324). Nearly 15% of all COVID-19 patients and pneumonia develop ARDS, a common consequence of the cytokine storm in the lung tissue and in systemic circulation (325). In such patients, predominant pulmonary pathologies in the lung tissue, which include low blood oxygen levels, diffuse alveolar damage, alveolar edema, and proteinaceous exudation, alveolar wall thickening, evident pneumocyte desquamation, and hyaline membrane formation (114, 311, 326), provide indicators for ARDS. Damaged alveolar epithelial cells and endothelial cells, and extensive phlegm secretion and exudation can trigger coagulation, hyperfibrinolysis, and small blood vessel thrombosis. They can also inhibit lung ventilation significantly (66), thereby increasing hypoxemia, hypotension and shock, promoting pulmonary embolism, and further increasing the severity of the disease (83, 327–330). Hence, ARDS-induced low oxygen saturation and respiratory failure are major causes of mortality in COVID-19 cases; they are the cause of death in 70% of all fatal COVID-19 cases (328, 330).

The factors that drive severe lung pathology during infection with highly pathogenic human coronaviruses are poorly understood. Computed tomography images revealed that COVID-19-infected lungs accumulate fluid-filled white patches called “ground-glass opacities” (303); however, the nature of the clear jelly remains to be determined. Potential mechanisms include high rates of viral replication that could be responsible for enhanced host cell cytolysis and the strong production of inflammatory cytokines and chemokines by infected epithelial cells and the delayed induction of antiviral IFN responses owing to virus escape mechanisms such as the production of IFN inhibitory proteins that perpetuate viral damage and lead to the excessive accumulation of monocytes, macrophages, and neutrophils (6, 302).

Susceptibility to SARS-CoV-2 infection is not age-dependent (median age at infection is approximately 50 years). However, evidence suggests that clinical severity differs with age (33, 71, 328, 331, 332). In general, compared with young people, children are either asymptomatic or they suffer only from mild pneumonia, whereas older men (>60 years old) with comorbidities are more likely to require intensive treatment, develop severe respiratory disease, or even die (28, 304, 331, 332). Moreover, men with COVID-19 tend to exhibit more severe morbidity and mortality than women (321, 333), and meta-analyses have shown that men with COVID-19 have threefold higher odds of requiring hospitalization and 2.4-fold higher mortality rate (321, 334).

### Extrapulmonary Manifestations

#### Neurological Manifestations

Coronaviruses primarily target the human respiratory system. However, COVID-19 infection has also been reported to trigger neurological manifestations and has neuroinvasive capabilities that permit its spread from the respiratory tract *via* the olfactory bulb, causing inflammation and demyelination in the central nervous system (CNS) (335, 336). Owing to the ACE2 receptors in olfactory cilial cells, the virus could potentially reach the CSF within seven days (325), while additional studies suggest that the virus could enhance inflammatory cytokines, including TNF-α and IL-6, leading to structural and metabolic damage in the CNS. Moreover, the levels of IL-8, CCL2, CCL3, CCL4, CCL7, CCL12, and CX3CL1 were higher in both the serum and CSF samples of COVID-19 cases with neurological syndrome (NS) (299).

More recent studies demonstrate that the epithelial cells of the blood-cerebrospinal fluid barrier (BCSFB) in the choroid plexus of the ventricles can act as a conduit for SARS-CoV-2 into the CNS (337, 338), leading to inflammation and disruption of the BCSFB integrity (339). The vulnerability to the CNS can result in exposure to complications such as neuroinflammation and encephalitis (340). The inflammation further upregulates the expression of CCL2, IL-1, IL-6, TNF-α, MMP-8, and ICAM-1, increasing the infiltration of inflammatory immune cells, such as monocytes, and enhancing the permeability of the BCSFB (290–292). In addition, CD4^+^ cells secreting cytokine IL-6, along with mast cells secreting cytokines and chemokines (including IL-1β, TNF-α, CCL2, CXCL10, and GM-CSF) accelerate neurodegeneration, cognitive dysfunction, and stroke (293, 326). As a result, approximately 36% of all patients with COVID-19 have neurological manifestations (312).

Neurological symptoms, involving the central/peripheral nervous system, have been predominantly reported in adults; however, children also experience various neurological insults owing to COVID-19 (341). For example, 28% of pediatric patients in the United States experienced headaches (342), and some developed febrile seizures (335, 343). In adults, neurological manifestations range from mild symptoms, such as headache, dizziness, anorexia, anosmia, myalgia/fatigue, and ageusia (33, 303, 328), to severe manifestations, including acute stroke (344, 345), confusion, or altered consciousness (343, 346), acute inflammatory demyelinating polyneuropathy (Guillain-Barré syndrome) (107, 347), meningoencephalitis, and acute necrotizing encephalopathy of the brainstem and basal ganglia (348, 349). While the cause and correlation of the neurological manifestations of COVID-19 have not been appropriately established, long-term effects on the nervous system deserve further investigation (335). Similar to the respiratory and cardiac manifestations of COVID-19, neurological complications present differently on the basis of age and underlying comorbidities (341).

#### Cardiovascular Manifestations

Cardiac manifestations have been reported in patients with no clinical features of respiratory disease, and patients with preexisting cardiac-related ailments, such as dyslipidemia, obesity, and diabetes, carry a greater risk of developing severe COVID-19 (350). The severity and lethality of COVID-19-associated cardiac dysfunction are attributed to viral-induced damage to the heart and blood vessels (337). Infection with the SARS-CoV-2 virus directly infects cardiomyocytes to damage the myocardium, and it induces T cell activation. This further increases IL-1, IL-6, and TNF-α secretions (338), and it contributes to COVID-19 post-infective acute myocarditis. In addition, an inclusion can form in the myocardium when SARS-CoV-2 particles are surrounded by pro-inflammatory cells, such as neutrophils, macrophages, and lymphocytes. The inflammatory cells release cytokines such as IL-1β, TNF-α, and CCL2, which are noxious factors to the heart contributing to hypoxia and shock (351). Subsequently, the heightened metabolic rate elevates the demand for myocardial oxygen, whereby hypoxia, respiratory distress, metabolic acidosis, and fluid or electrolyte disturbances are triggered (351, 352). In severe cases, activated neurohumoral systems may trigger cardiac arrest and damage as well as even induce myocarditis, myocardial infarction, and malignant arrhythmias (66, 83, 339, 340, 353–355). Multiple retrospective reports have suggested that in China, cardiovascular disease incidence ranges from 5% to 16%, hypertension incidence ranges from 15% to 31%, and coronary artery disease incidence is 11% (356, 357). Other countries have reported even higher incidence rates of these comorbidities (356).

#### Renal Manifestations

Acute kidney injury (AKI) is also frequently observed in patients with COVID-19, and its manifestations include hypotension, microvascular damage and contraction, decreased renal perfusion, and hemostasis, and related sepsis (13, 66). In the kidneys, SARS-CoV-2 may infect ACE2-expressing cells, like proximal tubules and podocytes, to accumulate Ang II and further promote an imbalanced RAS. This may result in glomerular dysfunction, fibrosis, vasoconstriction, and inflammation (358). Several studies also illustrated the importance of cytokines, which can interact with kidney-resident cells and induce endothelial and tubular dysfunction. They include IL-6, IL-1β, IL-1RA, IL-7, IL-8, IL-9, IL-10, fibroblast growth factor (FGF), GM-CSF, IFN-γ, G-CSF, CXCL10, CCL2, CCL3, PDGF, TNF-α, and VEGF (33, 89, 123, 359). In particular, elevated IL-6 would cause renal endothelial cells to secrete pro-inflammatory chemokines like CCL14 and CCL2, leading to kidney vascular permeability (360, 361). Moreover, IFNs could cause the loss of podocytes and stimulate glomerulosclerosis to promote AKI persistence (362). One autopsy report has indicated that renal pathology in COVID-19 shows podocyte damage, significant acute proximal tubule injury, and coronavirus particle clusters in podocytes and renal tubular epithelial cells (363).

#### Hepatobiliary Manifestations

Liver damage in COVID-19 can be directly caused by the viral infection of liver cells, and hepatic injury is, therefore, associated with the degree of disease severity (364). Mechanisms causing liver injury in patients with COVID-19 remain unclear; however, multiple theories have been postulated: (1) ACE2-mediated direct viral infection of gastrointestinal epithelial cells to dysregulate liver functions (351); (2) critical patient status and immune-mediated injuries, such as cytokine storm or pneumonia-associated hypoxia, or IL-6-mediated activation of the complement system and consequent increase in vascular permeability (364); and (3) drug hepatotoxicity (352).

As reported, given the higher ACE2 expression of cholangiocytes as compared to the population of hepatocytes (59.7% *vs*. 2.6%), SARS-CoV-2 uses ACE2 to gain entry in the cells (351, 365). Cholangiocytes are correlated with liver physiology and adaptive immune response mechanisms. Thus, the impairment of cholangiocyte function would result in hepatobiliary damage and disrupt the barrier and bile-acid-transporting functions (366) to further contribute to SARS-CoV-2-related liver injury (367). Second, an exaggerated hyperinflammatory response from significantly elevated C-reactive protein (CRP), LDH, ferritin, and IL-6 levels contributes to liver injury or even develops into liver failure in critically ill patients (364). After the entry of the virus, pathogenic T cells are activated rapidly, producing GM-CSF, IL-6, and IFN-γ. GM-CSF further induces CD14^+^CD16^+^ inflammatory monocytes to produce increased IL-6 (368, 369). This could activate more macrophages and contribute to an inflammatory storm (251, 368, 369). This immune-mediated injury, accompanied by elevated concentrations of IL-1, IL-2, IL-10, and IFNγ, would lead to systemic inflammatory response syndrome (SIRS) (368, 369). As for drug toxicity, it is possible that impairment to the liver is caused by drug hepatotoxicity. This might explain the large variations observed across different cohorts (370).

Multiple reports have indicated that some COVID-19 patients have an abnormal liver function; e.g., the American College of Gastroenterology reported that approximately 20%-30% of patients had elevated liver enzymes, and another study from China suggested that 50.7% of SARS-CoV-2-infected patients had liver abnormalities (371). Of note, elevated liver function results were reported to be associated with moderate-high degree fever and lower T cell function (both CD4^+^ and CD8^+^ T cells) (371), and patients with preexisting liver disease (such as hepatitis B infection) carry a higher risk of developing severe disease (372, 373).

### Multisystem Inflammatory Syndrome in Children (MIS-C)

Despite initial optimism with respect to children being spared the worst effects of COVID-19, it is now clear that they can also develop severe COVID-19 symptoms and a rare secondary inflammatory syndrome: multisystem inflammatory syndrome in children (MIS-C) (374). MIS-C was first identified in Northern Italy, followed by the UK, and is described as a post-COVID-19 inflammatory syndrome that is approximately 30-fold more common in the COVID-19 cohort than in healthy children (375). MIS-C symptoms range from mild, e.g., fever, rash, and mucocutaneous inflammation, to severe, including vasculitis, cardiac dysfunction, shock, and neurological involvement (376). This post-COVID-19 inflammatory syndrome was originally diagnosed as Kawasaki disease; however, patients with MIS-C display more pronounced lymphopenia, thrombocytopenia, anemia, and elevated serum IFN-γ, IL-1β, IL-6, IL-10, and IL-17 levels (377, 378). However, these effects are not as severe as those seen in severe cytokine storm syndromes, and while increased IL-10 level is correlated with a lower viral load (99), its presence, along with elevated TNF levels, is the optimal marker for distinguishing MIS-C from severe COVID-19 symptoms (379).

Similar to the neuroinvasive capabilities of SARS-CoV-2 and its CNS sequelae observed in adults, children with MIS-C also develop neurological phenomena along with respiratory symptoms and multisystem inflammation (341). Moreover, multiple studies have described neurological symptoms ranging from mild (headache and altered mental status) (380–382) to severe neurological complications, including seizure, coma, encephalitis, demyelinating disorders, encephalopathy, aseptic meningitis dysarthria, dysphagia, cerebellar ataxia, and peripheral neuropathy; the latter leads to global proximal muscle weakness and reduced reflexes (383).

## Discussions and Perspectives

The present review describes significant cytokine and chemokine secretions induced by innate and adaptive immune responses in COVID-19 and virus-associated immunopathogenesis. Available data from basic and clinical studies on critical cytokines and chemokines have been presented in this review in addition to a summary of pulmonary and extrapulmonary pathophysiological features of cytokine release syndrome, consequent systemic immune response to SARS-CoV-2, and potential immunotherapeutic approaches. This review suggests that understanding the nature of the COVID-19-induced cytokine/chemokine profile(s) and pathophysiological features of correspondent immune response to control excessive inflammatory response is as important as targeting the virus itself as fatalities are primarily caused by abnormal and exaggerated cytokine storms. Future research should focus on identifying cytokine biomarkers to define the immune correlates of protection and disease severity for the effective triage of patients.

## Author Contributions

Conception and design: R-JH and S-HY. Provision of study materials: W-CY, G-RP, C-HY, SH, and PC. Collection and assembly of data: KY, JL, and W-CL. Manuscript writing: All authors. Accountable for all aspects of the work: All authors. All authors contributed to the article and approved the submitted version.

## Funding

This work was financially supported by the Young Scholar Fellowship Program by Ministry of Science and Technology (MOST) in Taiwan, under Grant MOST 110-2636-B-002-022 and the Hualien Tzu Chi Hospital, Buddhist Tzu Chi Medical Foundation in Taiwan, under Grant TCRD111-075.

## Conflict of Interest

The authors declare that the research was conducted in the absence of any commercial or financial relationships that could be construed as a potential conflict of interest.

## Publisher’s Note

All claims expressed in this article are solely those of the authors and do not necessarily represent those of their affiliated organizations, or those of the publisher, the editors and the reviewers. Any product that may be evaluated in this article, or claim that may be made by its manufacturer, is not guaranteed or endorsed by the publisher.
